# *Dictyostelium*: An Important Source of Structural and Functional Diversity in Drug Discovery

**DOI:** 10.3390/cells8010006

**Published:** 2018-12-21

**Authors:** Yuzuru Kubohara, Haruhisa Kikuchi

**Affiliations:** 1 Laboratory of Health and Life Science, Graduate School of Health and Sports Science, Juntendo University, Inzai, Chiba 270-1695, Japan; 2 Laboratory of Natural Product Chemistry, Graduate School of Pharmaceutical Sciences, Tohoku University, 6-3 Aza-aoba, Aramaki, Aoba-ku, Sendai 980-8578, Japan; hal@mail.pharm.tohoku.ac.jp

**Keywords:** *Dictyostelium*, *Polysphondylium*, cellular slime mold, DIF, polyketide, drug resource, mitochondria, cancer, diabetes, *Trypanosoma cruzi*

## Abstract

The cellular slime mold *Dictyostelium discoideum* is an excellent model organism for the study of cell and developmental biology because of its simple life cycle and ease of use. Recent findings suggest that *Dictyostelium* and possibly other genera of cellular slime molds, are potential sources of novel lead compounds for pharmacological and medical research. In this review, we present supporting evidence that cellular slime molds are an untapped source of lead compounds by examining the discovery and functions of polyketide differentiation-inducing factor-1, a compound that was originally isolated as an inducer of stalk-cell differentiation in *D. discoideum* and, together with its derivatives, is now a promising lead compound for drug discovery in several areas. We also review other novel compounds, including secondary metabolites, that have been isolated from cellular slime molds.

## 1. Introduction

Natural products have been used as medicines and for drug development since ancient times and natural product chemistry remains important in the fields of drug discovery, structure elucidation and chemical synthesis. Among the current sources of lead compounds for drug discovery, microorganisms such as the fungi ascomycetes, basidiomycetes and deuteromycetes and the bacteria actinomycetes, have provided many useful drugs (e.g., antibiotics) [1,2,3]. Currently, in the field of medicine there are several major issues that need to be addressed, such as the development of drugs with improved adverse effect profiles; drugs to treat currently incurable diseases; and drugs against refractory bacteria, protozoans and cancer cells [1,2,3,4]. Thus, novel sources of lead compounds are needed.

**Figure 1:**
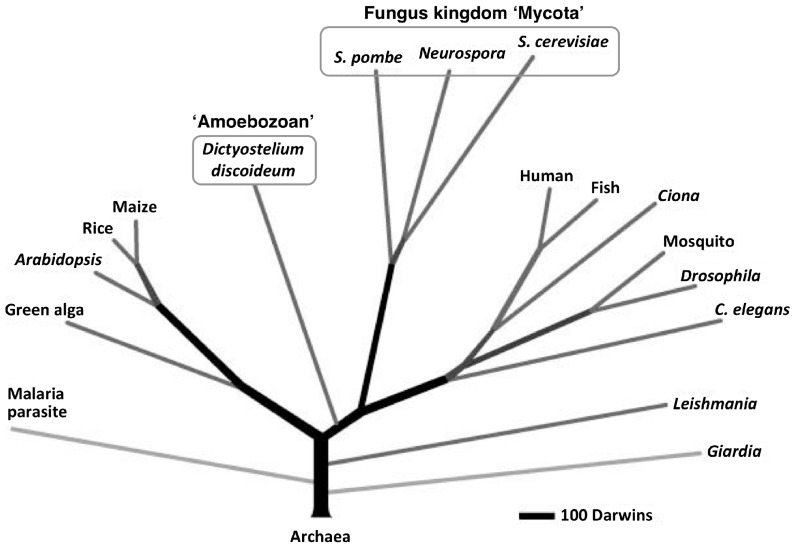
Proteome-based eukaryotic phylogeny (adapted with permission from Eichinger et al. [6]). The phylogenetic tree was constructed from a database of 5279 orthologous protein clusters that were drawn from 17 eukaryotic proteomes, including that of *Dictyostelium discoideum*, which was rooted on 159 protein clusters that had representatives from six archaebacterial proteomes: *Plasmodium falciparum*, malaria parasite; *Chlamydomonas reinhardtii*, green alga; *Oryza sativa*, rice; *Zea mays*, maize; *Fugu rubripes*, fish; *Anopheles gambiae*, mosquito.

The cellular slime molds are a group of soil microorganisms that belong to the eukaryotic kingdom Amoebozoa, which, according to recent taxonomic research, is distinct from the fungus kingdom Mycota (Figure 1) [5,6,7]. For about 80 years, the cellular slime mold *Dictyostelium discoideum* has been used as a model organism for the study of eukaryotic cell functions (e.g., division, differentiation, chemotaxis, autophagy and death) [8,9,10,11,12,13,14,15,16,17,18,19] mainly because of its simple life cycle and ease of handling. Recently, *D. discoideum* has also been used as a model organism for the study of human diseases and estimation of drug effects [17,20,21,22,23,24]. (See the other reviews in this special issue). Our group has been examining the use of cellular slime molds as a source of natural compounds and we have isolated several novel biologically significant compounds from several species of cellular slime molds [25,26,27,28,29,30,31,32,33,34].

Genome analyses of *Dictyostelium* cellular slime molds have revealed that *D. discoideum* has approximately 43 polyketide synthase genes [6] and that *D. purpureum* has 50 predicted polyketide synthase genes [35]. These numbers of polyketide synthase genes are greater than those in *Streptomyces avermitilis*, which is a bacterium known to produce many secondary metabolites; here, secondary metabolites are organic compounds biosynthesized from primary metabolites by taxonomically restricted spectrum of organism and not directly necessary for their growth and reproduction. This suggests that *Dictyostelium* cellular slime molds and possibly other genera of cellular slime molds [30,32], also produce an abundance of secondary metabolites that could be used as novel lead compounds for drug discovery.

Among the data on the candidate lead compounds our group has reported to date, we have made most progress regarding elucidation of the biological and pharmacological activities of the *D. discoideum* differentiation-inducing factors.

## 2. Biological and Pharmacological Activities of DIF-1 and Its Derivatives

### 2.1. Functions of DIF-1, DIF-2 and DIF-3 in D. discoideum

**Figure 2:**
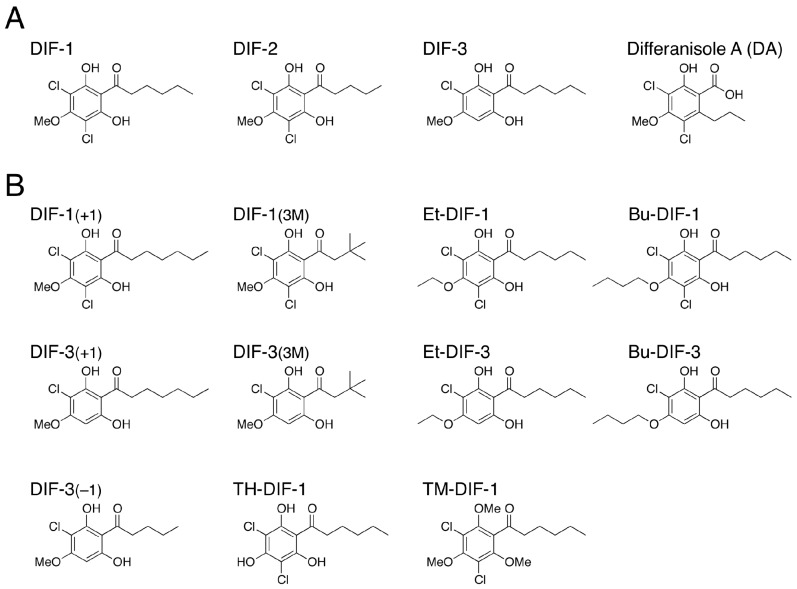
(**A**) Chemical structures of DIFs 1–3 and differanisole A. The order of the stalk-cell differentiation-inducing activity in *D. discoideum* in vitro is DIF-1 > DIF-2 >> DIF-3 [39,40]; (**B**) Chemical structures of 11 representative DIF derivatives.

DIF-1 (differentiation-inducing factor 1), DIF-2 and DIF-3 (Figure 2A) are chlorinated alkylphenones that were originally isolated from *D. discoideum* as inducers of stalk-cell differentiation [36,37]. Of the three compounds, DIF-1 is the most active so that DIF-1 at nanomolar levels dose-dependently induces stalk-cell differentiation in vitro; DIF-2 has only around 40% of the specific activity of DIF-1 [37,38,39,40] and DIF-3 has only around 4% of the activity of DIF-1 [40,41], although DIF-3 is the initial metabolite of DIF-1 in vivo [40,42]. Stalk cell differentiation is a sort of programmed cell death [43] and can be categorized as a type of autophagic cell death [11,44]. Therefore, DIF-1-induced stalk-cell differentiation is a good model system for the study of autophagy, autophagic cell death and programmed cell death [45,46,47].

In addition to having differentiation-inducing activities, DIFs 1 and 2 at nanomolar levels function as modulators for *Dictyostelium* chemotactic cell movement toward cyclic adenosine monophosphate (cAMP) [48,49]. Importantly, the mechanisms for the modulation of chemotaxis by DIFs differ, at least in part, from those for the induction of stalk-cell differentiation [48,49,50]. Since the discovery of DIFs 1 and 2, the mechanisms underlying their functions have been examined [11,41,44,45,46,47,48,49,50,51,52,53,54,55,56,57] but remain to be fully elucidated; most importantly, their receptors have not been determined.

It is important to note that DIFs 1 and 2 are endogenous polyketide factors in *D. discoideum* and DIF-3 is a metabolite [40,42,58]; they were not identified as drugs against human diseases such as antibiotics at first.

### 2.2. Discovery of the Antitumor Activities of DIFs

Two years before the discovery of DIF-1, Oka et al. [59] isolated a compound called differanisole A (DA) (Figure 2A) from the fungus *Chaetomium* (RB-001). DA induces growth arrest and re-differentiation of mouse erythroleukemia (B8) cells into hemoglobin-producing cells. On the basis of the structural similarity of DIF-1 and DA, it has been shown that DA (at high enough concentration) has the same effects as DIF-1 in *D. discoideum* [60], and, conversely, that DIF-1 at micromolar levels induces growth arrest and re-differentiation of mouse B8 cells into hemoglobin-producing cells in a dose-dependent manner [61]. Since the antitumor activity of DIF-1 is slightly higher than that of DA (unpublished observation), our group started to develop antitumor agents, utilizing DIF-1.

DIFs 1 and 3—especially DIF-3—have strong anti-proliferative activity and induce or promote cell differentiation in various mammalian tumor cell lines in vitro, including human leukemia K562 cells, human myeloid leukemia HL-60 cells, human gastric cancer cells and human cervical cancer HeLa cells [61,62,63,64,65,66]. In addition, under certain conditions (e.g., at high concentrations), DIFs 1 and 3 can induce cell death [67,68,69]. Note that the anti-proliferative and differentiation-inducing effects of DIFs are not limited to transformed cells (see Section 2.4.1) [66,70,71,72]; however, the anti-proliferative effect of DIFs in transformed cells is stronger than that in mouse 3T3-L1 fibroblasts (a model non-transformed cell) [71,72].

**Figure 3:**
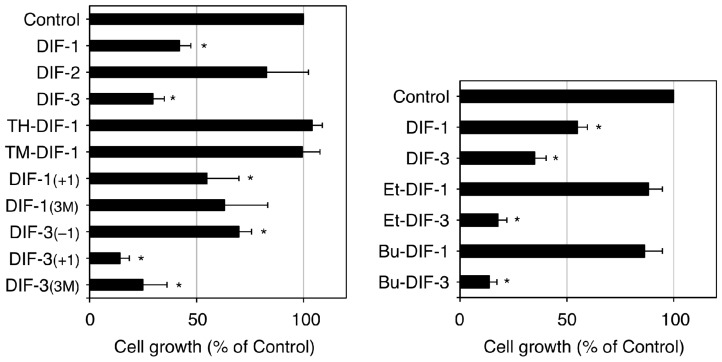
Effects of DIFs on the growth of K562 human leukemia cells (adapted from Gokan et al. [73]). Cells were incubated at 37 °C for 3 days in the presence of 0.15% EtOH (vehicle; Control) or 15 µM of one of the DIF derivatives and then the relative cell number was assessed. Means and SD (bars) of three independent experiments are shown. * *p* < 0.05 versus Control (by *t*-test; two-tailed, unpaired). DIF-3 and its derivatives but not DIF-1 and its derivatives, showed strong anti-proliferative activity in K562 cells.

Our group has investigated the chemical structure–activity relationship of more than 30 chemically synthesized DIF derivatives (Figure 2B) and has found several DIF-3 derivatives (e.g., DIF-3_(+1)_ and Bu-DIF-3) that are potent suppressors of cell growth and are therefore promising compounds for the development of anti-cancer drugs (Figure 3) [72,73,74,75].

**Figure 4:**
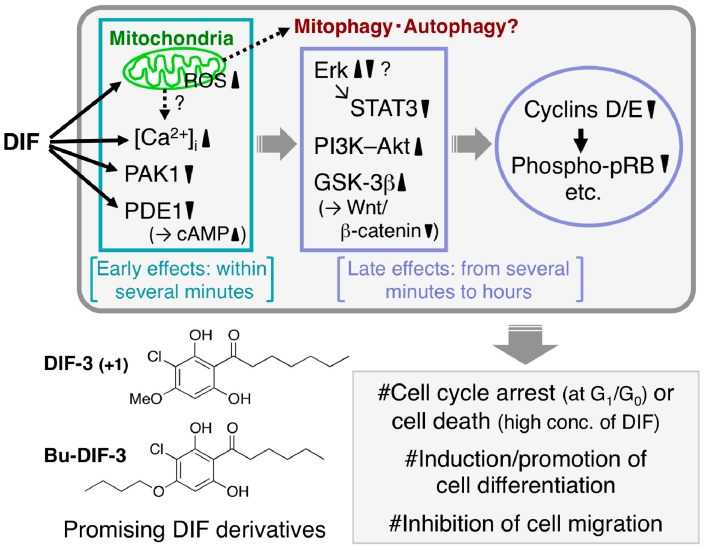
Proposed scheme of the antitumor effects of DIFs. After the addition of one of the DIFs to tumor cells, the DIF rapidly (within several minutes) disturbs mitochondrial function [69,77], increases reactive oxygen species (ROS) production [69] and intracellular calcium concentration ([Ca^2+^]_i_) [63,64,69,77] and inhibits the activities of p21-activated kinase 1 (PAK1) [74] and calmodulin-dependent cAMP/cGMP phosphodiesterase (PDE1) (resulting in an increase in cAMP levels) [76]. Over time (from several minutes to hours) the DIF also affects the activities of extracellular signal-regulated kinase (Erk), signal transducer and activator of transcription 3 (STAT3), phosphatidylinositol 3-kinase (PI3K)–Akt, glycogen synthase kinase-3β (GSK-3β) and the Wnt/β-catenin pathway, which suppresses the expression of cyclins D/E (and promotes the degradation of cyclin D1) and the subsequent reduction of phospho-pRB [65,66,74,77,78,79,80,81]. At appropriate concentrations the DIFs have been found to induce growth arrest in all the tumor cell lines tested to date in vitro and in vivo and at higher concentrations they have induced caspase-independent cell death [67,68,69]. Also, the DIFs induce differentiation of murine and human leukemia (B8 and K562) cells in vitro [61,64] and promote retinoic acid-induced differentiation of human leukemia HL-60 cells in vitro [63]. In addition, the DIFs suppress the migration of some cancer cells in vitro and in vivo [72,82]. Chemical structure–activity relationship analyses have revealed that DIF-3_(+1)_ and Bu-DIF-3 are promising lead compounds for the development of anti-cancer drugs [72,73,74].

The mechanisms underlying the antitumor activities of DIFs 1 and 3 and their derivatives have been partially elucidated (Figure 4); for example, it has been reported that (1) they rapidly increase intracellular calcium concentration in several tumor cell lines [62,63,64,68,69]; (2) they directly inhibit the activities of calmodulin-dependent cAMP/cGMP phosphodiesterase (PDE1) [76] and p21-activated kinase 1 (PAK1) [74]; (3) they function as mitochondrial uncouplers and disrupt mitochondrial functions, possibly resulting in the induction of mitophagy and autophagy [69,77]; (4) they affect the activities of several crucial enzymes such as phosphatidylinositol 3-kinase (PI3K) and Akt kinase (protein kinase B) [78], extracellular signal-regulated kinase (Erk) [65,79] and glycogen synthase kinase-3β (GSK-3β) [66,80] in several tumor cell lines; and (5) they suppress the expression of cyclins D/E and reduce the phosphorylation of retinoblastoma protein (pRB), resulting in cell-cycle arrest at the G_1_/G_0_ phase [70,79,81].

Recent studies have revealed that the DIFs inhibit cell migration in certain malignant cancer cell lines, such as mouse osteosarcoma LM8 cells and mouse (B16BL6) and human (A2058) melanoma cells, in vitro and in vivo (in mice) [72,82]. Also, DIF-3 inhibits intestinal tumor growth in vitro and in vivo (in mice) [83] and imatinib-resistant K562 leukemia cell growth in xenografted mice [69]. These observations suggest that DIFs have therapeutic potential for the treatment of malignant metastatic and drug-resistant cancers.

In the future, our group intends to elucidate the mechanisms underlying the actions of DIFs and develop DIF derivatives with more potent activities for use as lead compounds in anti-cancer drug discovery.

### 2.3. Glucose Uptake-Promoting Activity of DIF-1

**Figure 5:**
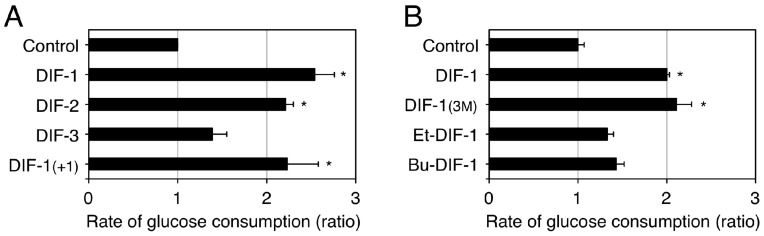
Effects of DIFs on glucose consumption (uptake) by confluent 3T3-L1 fibroblasts. Confluent 3T3-L1 fibroblasts were incubated at 37 °C for at least 8 h in the presence of 0.2% EtOH (vehicle; Control) or 20 µM of one of the DIF derivatives. The glucose concentration in each incubation medium was measured and the approximate rate of glucose consumption was calculated relative to that in the Control medium; the rate of glucose consumption corresponds well with that of tritium-labeled 2-deoxy-glucose uptake promoted by DIF-1 [84]. The means and SD (bars) of three independent experiments (**A**) or triplicate experiments (**B**) are shown (adapted from Omata et al. [84] and Kubohara et al. [71], respectively). * *p* < 0.05 versus Control (by *t*-test; two-tailed, unpaired).

As the first step to assessing the potential of using DIF-1 as a lead compound for anti-cancer drug development, our group investigated the toxic effects of DIF-1 in vitro by using confluent mouse 3T3-L1 fibroblasts and rat gastric mucosal RGM-1 cells, which are model non-transformed cell lines [84]. DIF-1 at 5–20 µM dose-dependently promoted glucose uptake without affecting cell morphology and cell number in the confluent 3T3-L1 fibroblasts and RGM-1 cells and also in 3T3-L1 adipocytes [84]. Chemical structure–activity relationship analysis revealed that some DIF derivatives, such as DIF-1 and DIF-1_(3M)_, increased glucose uptake by two to three times in confluent 3T3-L1 fibroblasts in vitro (Figure 5) [71,84]. Since the glucose uptake-promoting activity of DIFs (Figure 5) is not necessarily correlated with their anti-proliferative activity (Figure 3), the mechanisms underlying the actions of DIFs in promoting glucose uptake should differ from those through which the compounds suppress tumor cell growth.

**Figure 6:**
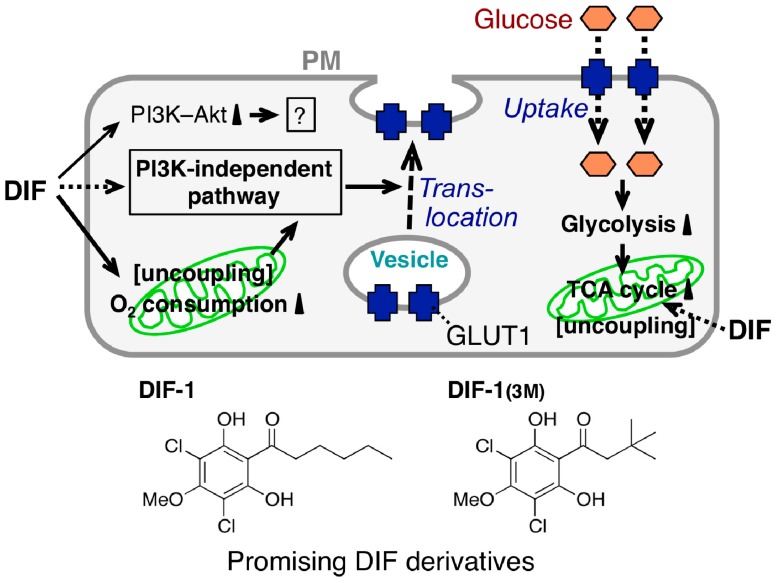
Proposed scheme for the glucose uptake-promoting effect of DIFs. Stimulation with a DIF induces glucose transporter 1 (GLUT1) translocation from intracellular vesicles to the plasma membrane (PM) via a PI3K–Akt-independent pathway, resulting in the promotion of glucose uptake by confluent mammalian cells [84]; the DIFs activate PI3K and Akt but this is not related to the DIF glucose uptake-promoting activity [84]. The DIFs also function as mitochondrial uncouplers, promoting oxygen consumption [77] and glucose metabolism (glycolysis and subsequent degradation in the tricarboxylic acid (TCA) cycle) [85] by mitochondria; this further increases GLUT1 translocation and promotes glucose uptake into the cells. Chemical structure-activity relationship analysis revealed that DIF-1 and DIF-1_(3M)_ are promising lead compounds for the development of anti-diabetes and anti-obesity drugs [71,84,85].

The mechanism underlying the glucose uptake-promoting activities of DIF-1 and DIF-1_(3M)_ has been partially elucidated (Figure 6); DIF-1 induces translocation of glucose transporter 1 (GLUT1) from intracellular vesicles to the plasma membrane via a PI3K–Akt-independent pathway, thereby promoting glucose uptake [84]. Note that DIF-1 (like insulin in 3T3-L1 adipocytes) was found to activate the PI3K–Akt pathway in all of the cell lines tested [84]. However, since DIF-1 promotes glucose uptake even in the presence of the PI3K inhibitors wortmannin and LY294002 in 3T3-L1 fibroblasts and 3T3-L1 adipocytes [84], the glucose uptake-promoting effect of DIF-1 is likely PI3K−Akt-independent. Also, DIF-1 and DIF-1_(3M)_ disturb mitochondrial activity, possibly by acting as uncouplers and promote cellular glucose metabolism in vitro [77,85]. During analysis of the antitumor activity of DIF-3, Dubois et al. [69] found that DIF-3 at 20 µM induces a loss of mitochondrial membrane potential, possibly by acting as an uncoupler and decreases cellular ATP levels in K562 leukemia cells. However, the glucose uptake-promoting activity of DIF-3 is considerably lower than those of DIF-1 and DIF-1_(3M)_ in 3T3-L1 fibroblasts (Figure 5) and neither DIF-1 nor DIF-1_(3M)_ at 20 µM significantly affects cellular ATP levels in 3T3-L1 fibroblasts [85]; our group is currently examining the mechanism underlying DIF-induced translocation of GLUT1.

In vivo analyses have shown that intraperitoneal injection of DIF-3_(3M)_ lowers blood glucose levels (after a meal) in KK-Ay diabetic mice [71] and that oral administration of DIF-1 lowers blood glucose levels in streptozotocin-induced diabetic rats without any apparent adverse effects [85]. These results suggest that DIF-1 and its derivatives may have therapeutic potential for the treatment of obesity and diabetes—especially of insulin-resistant diabetes.

### 2.4. Other Biological Activities of DIFs

#### 2.4.1. Differentiation-Inducing and -Promoting Activities

As already mentioned, DIFs 1 and 3 at 10–30 µM dose-dependently induce cell differentiation in vitro in murine (B8) and human (K562) leukemia cells [61,64]. Also, DIF-1 at low concentrations (1−5 µM) dose-dependently promotes retinoic acid-induced granulocyte differentiation in human HL-60 leukemia cells in vitro but it does not affect vitamin D-induced monocyte differentiation in HL-60 cells [63]. In addition, DIF-1 at 30 µM induces re-differentiation of de-differentiated vascular smooth muscle cells (non-transformed cells) isolated from human umbilical arteries in vitro [70]. Dimethyl sulfoxide at 1% (*v*/*v*) induces cardiomyocyte differentiation in vitro in P19CL6 embryonic carcinoma cells and the activity of dimethyl sulfoxide is promoted in the presence of Br-DIF-1, a chlorine-to-bromine substituted derivative of DIF-1, at 1–3 µM [86]. In contrast, DIF-1 at 5–30 µM suppresses osteoblast differentiation markers in human osteosarcoma SaOS-2 cells in vitro [87]. Together, these results suggest that DIFs could be useful as differentiation-inducing (or promoting) factors for obtaining various types of objective cells from embryonic or induced pluripotent stem (iPS) cells and as lead compounds for the development of anti-cancer chemotherapies.

#### 2.4.2. Anti-Meiotic Activity

*Xenopus* oocytes are a good model for investigating the mechanisms of meiosis and the associated signal transduction system. Maturation of *Xenopus* oocytes can be induced in vitro with progesterone, which subsequently induces germinal vehicle breakdown. DIF-1 at 10–40 µM dose-dependently suppresses progesterone-induced germinal vehicle breakdown in *Xenopus* oocytes in vitro, at least in part, by inhibiting a mitogen-activated protein kinase cascade [88].

#### 2.4.3. Immunomodulatory Activities in Jurkat T Cells

Since DIFs 1 and 3 and their derivatives have been shown to exhibit multiple biological activities in *D. discoideum*, *Xenopus* oocytes and mammalian cells, we hypothesized that DIF derivatives may have additional biological activities in other mammalian and eukaryotic cells.

**Figure 7:**
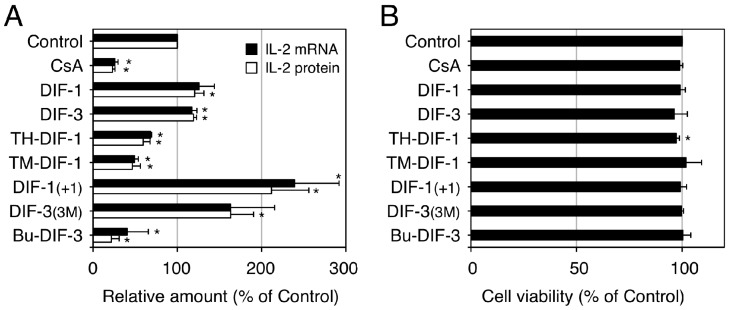
Effects of DIFs on ConA-induced IL-2 production in human Jurkat T cells. Jurkat T cells were pre-incubated at 37 °C for 0.5 h in the presence of 0.1% EtOH (vehicle; Control), 1 µM cyclosporin A (CsA), or 5 µM of one of the DIF derivatives. After the addition of ConA (as a mitogen), the cells were further incubated at 37 °C for 3 h and assayed for IL-2 mRNA expression (**A**), whereas another set of cells were incubated for 12 h (**A**,**B**) and assayed for IL-2 protein secretion (**A**) and for viability by using an MTT assay (**B**). The means and SD (bars) of three independent experiments are shown (adapted from Takahashi et al. [89]). * *p* < 0.05 versus Control (by *t*-test; two-tailed, unpaired). ConA-induced IL-2 production was significantly suppressed by the known immunosuppressive drug, CsA and by TH-DIF-1, TM-DIF-1 and Bu-DIF-3 but it was significantly promoted by DIF-1_(+1)_ and DIF-3_(3M)_; cell viability at 12 h was not affected by any of the compounds, except for TH-DIF-1.

We then investigated the effects of DIFs 1 and 3 and their derivatives on interleukin-2 (IL-2) production in vitro in human Jurkat T cells, a model cell line suitable for the study of T lymphocytes [89,90]. In Jurkat T cells, IL-2 production can be induced in vitro by stimulation with mitogens such as concanavalin A (ConA) and ConA-induced IL-2 production can be suppressed with the immunosuppressive drug cyclosporin A (CsA) (Figure 7). Our group found that some DIF derivatives, including TH-DIF-1, TM-DIF-1 and Bu-DIF-3, at low doses (e.g., 5 µM) significantly suppressed ConA-induced IL-2 production, whereas other DIF derivatives, including DIF-1_(+1)_ and DIF-3_(3M)_, significantly promoted ConA-induced IL-2 production in Jurkat T cells, with little effect on cell viability (Figure 7) [89,90]. Since IL-2 production in T cells is an index of immune system activity in vivo, these results suggest that DIF derivatives could be developed as novel immunosuppressive (and anti-inflammatory) or immunopromotive drugs.

#### 2.4.4. Anti-Trypanosoma Activity

*Trypanosoma cruzi* is the protozoan parasite that causes Chagas disease (human American trypanosomiasis). Despite the large number of deaths each year (>15,000) [91], therapeutic options for acute cases are limited (e.g., benznidazole and nifurtimox) [92,93] and there is no effective therapy for chronic cases.

**Figure 8:**
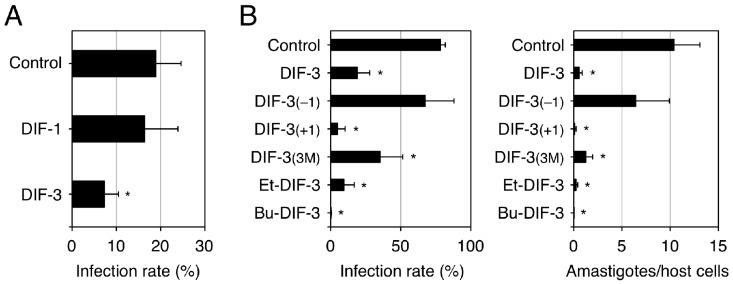
Effects of DIFs on the infection and growth of *Trypanosoma cruzi* in HT1080 cells. *Trypanosoma cruzi* (**A**: 1 × 10^5^ cells/well, **B**: 5 × 10^6^ cells/well) were incubated at 37 °C for 3 days in vitro with human fibrosarcoma HT1080 cells (host cells) in the presence of 0.1% EtOH (vehicle: Control) or 10 µM of one of the DIF derivatives. Then, the infection rate (parasite-infected HT1080 cells/total HT1080 cells) (**A**,**B**) and the number of amastigotes (intracellular form of *T. cruzi*) in the HT1080 cells (**B**) were assessed microscopically [94]. The means and SD (bars) of three independent experiments are shown (adapted from Nakajima-Shimada et al. [94]). * *p* < 0.05 versus Control (by *t*-test; two-tailed, unpaired). DIF-3 and some of its derivatives strongly suppressed *T. cruzi* infection and growth in the host cells.

To assess the pharmacological potential of DIFs 1 and 3 and their derivatives for the development of anti-*T. cruzi* drugs, our group examined the effects of these compounds on the infection rate and growth of *T. cruzi* in an in vitro assay system utilizing human fibrosarcoma HT1080 cells as host cells [94]. We found that DIF-3 derivatives such as DIF-3_(+1)_ and Bu-DIF-3 at 10 µM possessed strong anti-*T. cruzi* activities in vitro (Figure 8) and that intraperitoneally administered Bu-DIF-3 suppressed the increase in blood *T. cruzi* concentration in mice [94]. Interestingly, we also found that the DIF-3 derivatives that had strong anti-*T. cruzi* activity (Figure 8) also had strong anti-proliferative activity in tumor cells (Figure 3), suggesting that the activities of these derivatives in the two cell types may have similar underlying mechanisms.

#### 2.4.5. Anti-β-Amyloid Activity

Alzheimer’s disease is a form of dementia that is broadly characterized by memory loss and cognitive deterioration. During the progression of Alzheimer’s disease, extracellular plaques of β-amyloid and neurofibrillary tangles form in specific regions of the brain. Since β-amyloid is produced physiologically from amyloid-β protein precursor (APP) by most cells but particularly by neurons, it is thought that abnormal processing of APP in neurons results in the abnormal β-amyloid formation that characterizes Alzheimer’s disease [95,96].

Myre et al. [97] have shown that DIF-1 at 30 µM reduces amyloidogenic processing of APP in CHO-7W cells stably expressing human APP in vitro; this suggests that DIF-1 could be a novel anti-β-amyloid drug. However, since DIF-1 at 10–40 µM is toxic to rat cortical neurons in primary culture in vitro [98], clinical use of DIF-1 would likely have adverse effects associated with the cortical neurons. Further investigation into the effects of the other DIF derivatives on APP processing and neuronal functions is warranted. However, for now, DIF-1 is a promising lead compound for the development of anti-β-amyloid and thus anti-Alzheimer’s disease, drugs.

#### 2.4.6. Conclusions on the Activities of DIFs

**Figure 9:**
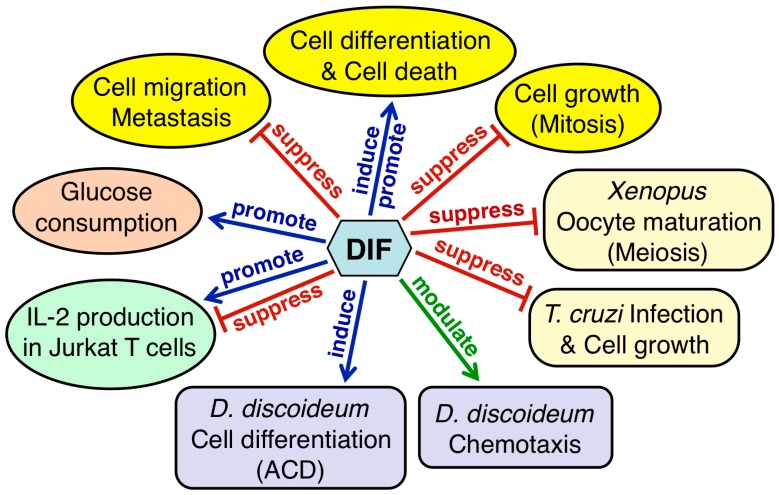
Summary of the physiological functions of DIFs 1 and 2 in *Dictyostelium discoideum* and the biological activities of DIF derivatives in other organisms. DIFs 1 and 2 function as inducers of stalk-cell differentiation (autophagic cell death: ACD) and as modulators of chemotactic cell movement in *D. discoideum* (purple rectangles). DIF derivatives have various biological activities in mammalian cells (yellow, orange and green ellipses) and in *Xenopus* oocytes and *Trypanosoma cruzi* (pale yellow rectangles).

Figure 9 summarizes the physiological functions of DIF-1 and DIF-2 in *D. discoideum* and the biological activities of DIF derivatives in other organisms. Considering that DIFs possess a range of biological activities in various eukaryotic cells, the DIFs and their derivatives likely have some undiscovered biological and pharmacological activities. Why DIFs possess such a range of biological activities is unknown; however, elucidation of the mechanisms underlying these activities—especially identification of the target molecules—will help in answering this question. Since DIFs possess various biological activities, there would be multiple target molecules of the DIFs; some candidate targets of DIFs that may be involved in the functions of DIFs have been reported (Table 1). Direct inhibition of PDE1 and PAK1 by DIFs may cause antitumor effects [74,76], whereas uncoupling of mitochondrial activity by DIFs may cause antitumor effects and/or promote glucose consumption in mammalian cells [72,77,85]. Matsuda et al. [99] have reported that DIF-1 but not DIF-3, directly inhibits mitochondrial malate dehydrogenase (mMDH), which may affect glucose consumption.

The data obtained to date strongly suggest that by modifying the side chains of the reported DIF derivatives we may be able to obtain compounds that have specific biological or pharmacological activities and that these compounds will be useful lead compounds for the development of anti-cancer, anti-obesity/diabetes, anti-*T. cruzi* and immunomodulatory agents.

The fact that DIF-like molecules such as DIFs 1–3 and DA (Figure 2) are produced by species in different kingdoms suggests that various DIF-like molecules are produced by all of the organisms belonging to Amoebozoa and Mycota. Furthermore, DA, which was found as an anti-tumor agent, may have a physiologic function, such as the induction of cell differentiation, in the organism in which it was first identified, namely *Chaetomium*.

## 3. Novel Biologically Active Compounds Found in Cellular Slime Molds

### 3.1. Dictyopyrones

**Figure 10:**
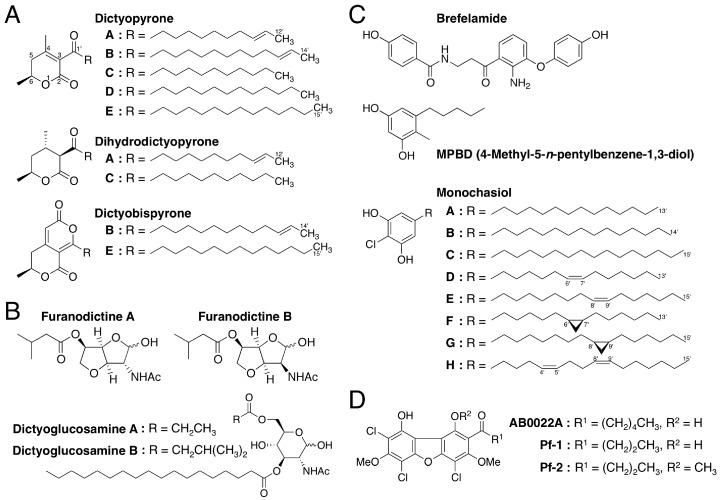
Chemical structures of secondary metabolites isolated from cellular slime molds. (**A**) dictyopyrones; (**B**) furanodictines; (**C**) Brefelamide, MPBD and Monochasiols; (**D**) AB0022A, Pf-1 and Pf-2.

While searching for biologically active secondary metabolites, Takaya et al. [100] isolated two novel α-pyronoids, dictyopyrones A and B (Figure 10A), from methanol extracts of the fruiting bodies of *D. discoideum* and *D. rhizoposium* and another α-pyronoid, dictyopyrone C (Figure 10A), from methanol extracts of the fruiting bodies of *D. longosporum*. Later, dictyopyrone A was also isolated from *D. longosporum*, dictyopyrone B was isolated from *D. magnum* and *D. mucoroides* and a new α-pyronoid, dictyopyrone D, was isolated from *D. magnum* (Figure 10A) [101]. Furthermore, Kikuchi et al. [29] isolated dihydrodictyopyrones A and C from *D. firmibasis* (Figure 10A). Although there are several known α-pyronoids with a hydroxyl group at the C-4 position, the dictyopyrones bear a unique α-pyrone moiety (3-acyl-4,6-dialkyl-α-pyrone ring) with a side chain at the C-3 position. This indicates that *Dictyostelium* cellular molds possess one or more unique biosynthetic pathways, providing further evidence that they are potentially valuable sources of lead compounds.

Recently, the production of dictyobispyrones B and E, alongside the production of dictyopyrones B and E, was induced in *D. giganteum* in the presence of zinc (II) ion (Figure 10A) [102]. The dictyobispyrones contain an α,α-bispyrone skeleton that can be biosynthesized from two distinct polyketide chains and therefore they could be biosynthetic precursors for the production of dictyopyrones through hydration and decarboxylation reactions.

By using chemically synthesized dictyopyrones [100,101], we have shown that dictyoypones A–D at micromolar levels promote morphogenesis of *D. discoideum* [100,101] and that dictyopyrones A and B at micromolar levels inhibit spore formation and promote stalk-cell formation in vitro in *D. discoideum* [103]. Also, we have reported that dictyopyrones and their derivatives suppress cell growth in human leukemia K562 cells in vitro [101].

### 3.2. Amino Sugar Derivatives: Furanodictines and Dictyoglucosamines

Kikuchi et al. [25] isolated two novel amino sugar derivatives, furanodictines A and B (Figure 10B), from methanol extracts of the fruiting bodies of *D. discoideum.* These compounds are derivatives of *N*-acetylglucosamine and *N*-acetylmannosamine, respectively and were the first 3,6-anhydrosugars isolated from a natural product. The unique structures of the furanodictines are intriguing and four research groups have reported four different synthetic pathways [25,104,105,106].

Two other amino sugar derivatives, dictyoglucosamines A and B (Figure 10B), were isolated from methanol extracts of *D. purpureum* and *D. discoideum*, respectively [26]. These compounds are characteristic in that the amino sugar is connected directly to the fatty acid.

The biological activities of these amino sugar derivatives were investigated and it was found that (1) furanodictine B but not furanodictine A, at 20 µM increases neurite formation in vitro in rat pheochromocytoma PC-12 cells, which are a model of neuronal differentiation; (2) furanodictine A and furanodictine B at 0.5–5 µM dose-dependently promote neurite formation in the presence of nerve growth factor (NGF) [25]; and (3) dictyoglucosamine A and dictyoglucosamine B at 1–10 µM dose-dependently induce neurite formation in PC-12 cells [26]. Thus, these amino sugar derivatives may be good lead compounds for the development of novel nerve-rejuvenation drugs for treating neurodegenerative diseases such as Alzheimer’s disease.

### 3.3. Brefelamide

Brefelamide (Figure 10C) is an aromatic amide that was isolated from methanol extracts of the fruiting bodies of *D. brefeldianum* and *D. giganteum* [27,107]. The 2-amino-3-hydroxy-β-aminopropiophenone moiety of brefelamide, which could be biosynthesized from tryptophan, is a rare structure in natural compounds.

Brefelamide at 1–100 µM dose-dependently suppresses cell growth in human astrocytoma 1321N1 cells in vitro through reduced glial cell line-derived neurotrophic factor (GDNF) receptor expression, reduced GDNF secretion and reduced phosphorylation of Erk, Akt and c-Jun N-terminal kinases [27,107]. Also, brefelamide at 12.5–50 µM suppresses the growth of and invasion by, A562 lung cancer cells in vitro, at least in part by inhibiting osteopontin expression [108]. In addition, brefelamide and its *O*-methyl derivative suppress osteopontin production in dengue serotype 3 virus-infected THP-1 cells, indicating that these compounds can prevent exacerbation of the illness to dengue hemorrhagic fever or dengue shock syndrome [109].

### 3.4. MPBD

MPBD (4-methyl-5-*n*-pentylbenzene-1,3-diol) (Figure 10C) is a polyketide that was isolated independently by two research groups as a secondary metabolite from *D. mucoroides* [28] and as an endogenous differentiation-inducing factor from *D. discoideum* [110]. Although the physiologic functions of MPBD in these organisms are unclear, MPBD at low nanomolar concentrations promotes both stalk-cell differentiation (albeit slightly) and spore differentiation in *D. discoideum* under some in vitro culture conditions [28,110]. At 20–80 µM, MPBD dose-dependently suppresses the growth of human leukemia K562 and HL-60 cells in vitro [28]. In addition, MPBD and its synthetic derivatives possess antimicrobial activities against *Escherichia coli* and *Bacillus subtilis* [111].

### 3.5. Monochasiols

Monochasiols A–H (Figure 10C) are chlorinated alkylresorcinols (and also polyketides) isolated from the fruiting bodies of *D. monochasioides* [33]. Although elucidation of their biological activities is ongoing, it has been shown that monochasiol A at 5–20 µM suppresses ConA-induced IL-2 production in Jurkat T cells without affecting cell viability [33].

Since the monochasiols can potentially be biogenetically synthesized by combining biosynthetic enzymes related to the principal polyketides DIF-1 and MPBD produced by *D. discoideum*, *Dictyostelium* cellular slime molds may produce a diverse range of monochasiol-based secondary metabolites.

### 3.6. Dibenzofurans: AB0022A, Pf-1 and Pf-2

AB0022A (Figure 10D) is an antimicrobial agent that is produced by *D. purpureum* and inhibits the growth of several Gram-positive but not Gram-negative, bacteria (minimal inhibitory concentration, 0.39–50 µg/mL; 0.85–109 µM) [112]. Recently, two other chlorinated dibenzofurans, Pf-1 and Pf-2 (Figure 10D), were isolated from the fruiting bodies of *Polysphondylium filamentosum* [32]. Although the antimicrobial activities of Pf-1 and Pf-2 are unknown, Pf-1 at 0.1–2 µM, like DIF-1, dose-dependently induces stalk-cell formation in *D. discoideum* in vitro, whereas AB0022A and Pf-2 at up to 2 µM do not [32]. AB0022A and Pf-1 but not Pf-2, at low micromolar concentrations suppress the growth of human leukemia K562 and HL-60 cells in vitro [32]. These findings suggest that, like DIF derivatives, the chlorinated dibenzofurans and their derivatives may possess multiple biological activities and that *Polysphondylium* species are also promising sources of lead compounds for natural product chemistry.

### 3.7. Prenylated and Geranylated Aromatic Compounds: Pt-1–5 and Ppc-1

**Figure 11:**
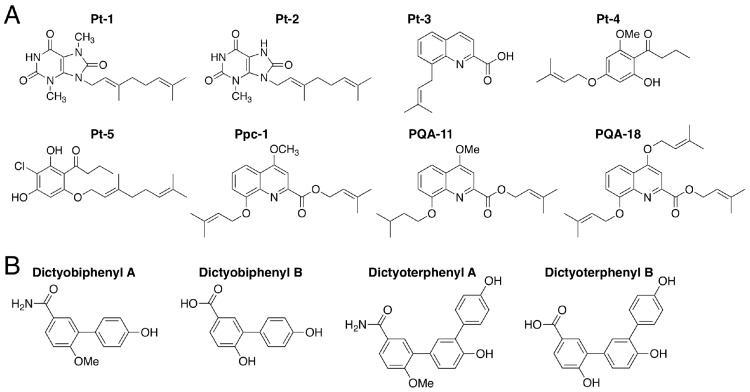
(**A**) Chemical structures of Pt-1–5 and Ppc-1 isolated from *Polysphondylim tenuissimum* and *P. pseudo-candidum* and two derivatives of Ppc-1, PQA-11 and PQA-18; (**B**) Chemical structures of dictyobiphenyls A and B and dictyoterphenyls A and B, isolated from *Dictyostelium polycephalum*.

The novel aromatic compounds Pt-1–5 and Ppc-1 (Figure 11A) were isolated from *Polysphondylium tenuissimum* and *P. pseudo-candidum*, respectively [30]. These compounds bear prenyl or geranyl groups. Pt-4 and Pt-5, which also each bear a butanoyl group, can be biosynthesized via the analogous polyketide DIF-1 (Figure 2A), which contains a hexanoyl group. The difference in length of the acyl groups may account for the chemotaxonomic differences between the genera *Dictyostelium* and *Polysphondylium*.

Pt-1, Pt-5 and Ppc-1 at 15 µM suppress the growth of human leukemia K562 cells in vitro and Ppc-1 suppresses the growth of HeLa cells in vitro [30]. Ppc-1 at 20 µM, like DIF-1, promotes glucose consumption by 3T3-1 cells in vitro [30] and intraperitoneally administered Ppc-1 induces weight loss in mice, possibly by uncoupling mitochondrial function [113]. Furthermore, Ppc-1 and its derivative, PQA-18 (Figure 11A), suppress IL-2 production in Jurkat T cells in vitro [114]. Another Ppc-1 derivative, PQA-11 (Figure 11A), has potent neuroprotective activities in vitro and in vivo, possibly via the inhibition of mitogen-activated protein kinase kinase 4 (MKK4) [115].

### 3.8. Dictyobiphenyls and Dictyoterphenyls

Dictyobiphenyls A and B and dictyoterphenyls A and B, are novel aromatic compounds (Figure 11B) that were isolated from the fruiting bodies of *D. polycephalum* [13]; note that dictyoterphenyl A was the first nitrogen-containing natural *m*-terphenyl isolated. Dictyoterphenyl A at 1–10 µM can suppress the growth of several cancer cell lines in vitro, such as the K562, HeLa and LM8 cell lines [13].

## 4. Conclusions

In this review, we have shown that DIFs 1 and 3 and their derivatives possess multiple biological activities in a variety of eukaryotic cells and the data suggest that they will be useful lead compounds for the development of anti-cancer, anti-obesity/diabetes, anti-*T. cruzi* and immunomodulatory agents. Moreover, our group has isolated various novel and unique compounds from *Dictyostelium* and *Polysphondylium* cellular slime molds and we have shown that some of these compounds have biological activities in mammalian cells in vitro and in vivo, which are summarized in Table 2. Together, the data strongly suggests that cellular slime molds are excellent sources of lead compounds for natural product chemistry and the development of next-generation drugs.

## 5. Patents

Patents related to our work on DIFs and other compounds:

Kubohara, Y.; Shibata, H. Agents that promote glucose metabolism and a method for screening anti-obesity and anti-diabetes drugs. Japanese Patent No. 4534039, 25 June 2010.

Kubohara, Y.; Shimada, J. Anti-*Trypanosoma* agents and drugs for trypanosomiases. Japanese Patent No. 5610433, 12 September 2014.

Kubohara, Y.; Murakami, M.; Takahashi, K.; Oshima, Y.; Kikuchi, H. Inhibitors of interleukin-2 production. Japanese Patent No. 5630751, 17 October 2014.

Kikuchi, H.; Oshima, Y.; Hattori, T.; Kubohara, Y.; Yamada, O.; Zhang, J.; Matsushita, Y.; Kida, S. Osteopontin production inhibitor with dictyopyrone or dihydrodictyopyrone derivatives as the active ingredient. Japanese Patent No. 5716140, 20 March 2015; Australian Patent No. 2013380489, 14 January 2016; Korean Patent No. 1593018, 2 February 2016; Canadian Patent No. 2896446, 28 June 2016; US Patent No. 9463188 B2, 11 October 2016; Chinese Patent No. ZL201380069437.4, 9 June 2017; EU Patent No. 2965758, 3 January 2018.

Honma, Y.; Suzuki, T.; Ogura, M; Oshima, Y.; Kikuchi, H. Prenyloxyquinoline carboxylic-acid derivative. Japanese Patent No. 6348845, 8 June 2018.

## Figures and Tables

**Table 1:** Targets of DIFs found in mammalian cells.

DIF Species Examined	Target of DIF	Biological Activities	Reference
DIF-1, DIF-3	PDE1	Direct inhibition of PDE1 activity	[76]
DIF-1	mMDH	Direct inhibition of mMDH activity	[100]
DIF-1, DIF-3, & their derivatives	Mitochondria	Uncoupling of mitochondrial activity	[72,77]
DIF-3 derivatives (e.g., DIF-3_(+1)_)	PAK1	Direct inhibition of PAK1 activity	[74]

**Table 2:** Origins and biological activities of the compounds that were found in cellular slime molds.

Compound	Source Organism	Biological Activities	Reference
Dictyopyrone A	*D. discoideum* *D. rhizoposium* *D. longosporum*	Promotion of morphogenesis & stalk cell differentiation, & inhibition of spore formation in *D. discoideum*	[100,101,103]
		Anti-proliferative activity in K562 cells	[101]
Dictyopyrone B	*D. discoideum* *D. rhizoposium* *D. magnum* *D. longosporum*	Promotion of morphogenesis & stalk cell differentiation, & inhibition of spore formation in *D. discoideum*	[100,101,103]
Dictyopyrone C	*D. longosporum*	Promotion of morphogenesis in *D. discoideum*	[100,101]
		Anti-proliferative activity in K562 cells	[101]
Dictyopyrone D	*D. magnum*	Promotion of morphogenesis in *D. discoideum*	[101]
Dihydrodictyopyrones A & C	*D. firmibasis*	N.D.	[29]
Dictyobispyrones B & E	*D. giganteum*	N.D.	[102]
Furanodictine A	*D. discoideum*	Promotion of NGF-induced neurite formation in PC-12 cells	[25]
Furanodictine B	*D. discoideum*	Induction of neurite formation in PC-12 cells	[25]
		Promotion of NGF-induced neurite formation in PC-12 cells	[25]
Dictyoglucosamine A	*D. purpureum*	Induction of neurite formation in PC-12 cells	[26]
Dictyoglucosamine B	*D. discoideum*	Induction of neurite formation in PC-12 cells	[26]
Brefelamide (& derivatives)	*D. brefeldianum* *D. giganteum*	Anti-proliferative activity in 1321N1 cells	[27,107]
		Anti-proliferative & anti-metastatic activities in A562 cells	[108]
		Inhibition of GDNF secretion in astrocytoma cells	[27,107]
		Anti-dengue viral activity	[109]
MPBD (& derivatives)	*D. discoideum* *D. mucoroides*	Promotion of cell differentiation in *D. discoideum*	[110]
		Anti-proliferative activity in K562 and HL-60 cells	[28]
		Antimicrobial activities vs. *E. coli* and *B. subtilis*	[111]
Monochasiol A	*D. monochasioides*	Inhibition of IL-2 production in Jurkat T cells	[33]
Monochasiols B–H	*D. monochasioides*	N.D.	[33]
AB0022A	*D. purpureum*	Antimicrobial activities vs. Gram-positive bacteria	[112]
Pf-1	*P. filamentosum*	Anti-proliferative activity in K562 and HL-60 cells	[32]
Pf-2	*P. filamentosum*	Stalk-cell-inducing activity in *D. discoideum*	[32]
Pt-1	*P. tenuissimum*	Anti-proliferative activity in K562 cells	[30]
Pt-2, Pt-3, Pt-4	*P. tenuissimum*	N.D.	[30]
Pt-5	*P. tenuissimum*	Anti-proliferative activity in K562 cells	[30]
Ppc-1 (& derivatives)	*P. pseudo-candidum*	Anti-proliferative activity in K562 and HeLa cells	[30]
		Promotion of glucose consumption in 3T3-L1 cells & mitochondrial uncoupling	[30,112]
		Inhibition of IL-2 production in Jurkat T cells	[114]
		Neuroprotective activities in vitro and in vivo	[115]
Dictyobiphenyls A & B	*D. polycephalum*	N.D.	[31]
Dictyoterphenyl A	*D. polycephalum*	Anti-proliferative activity in K562, HeLa and LM8 cells	[31]
Dictyoterphenyl B	*D. polycephalum*	N.D.	[31]

Footnote: N.D., not detected.
